# Retraction: miR-219-5p targets TBXT and inhibits breast cancer cell EMT and cell migration and invasion

**DOI:** 10.1042/BSR-2021-0318_RET

**Published:** 2024-11-11

**Keywords:** cell invasion, cell migration, EMT, miRNA-103, TBXT

This article “miR-219-5p targets TBXT and inhibits breast cancer cell EMT and cell migration and invasion” DOI: 10.1042/BSR20210318 is being retracted from *Bioscience Reports* at the request of the Editor-in-Chief and the Editorial Board. This follows receipt of a notification from a reader, alerting the Editorial Board to similarities between images in this paper. Specifically, between:

- Figure 2D MDA-MB-231/Mock and MDA-MB-231/miR-219-5p/HA-TBXT panels
- Figure 2G MDA-MB-231/NC Invasion panel and Figure 3B BT474/mIR-219-5p Invasion panel with colour alteration.

The authors were contacted regarding the concerns raised and provided repeat data for Figures 2D and 3B, however these images also appeared to contain some overlapping areas. Therefore, the issues with the published images were not resolved, and the journal has concerns surrounding the acquisition, labelling, and scientific validity of the data in this article. Dahu Chen and Qin Ye agree with the retraction. No response was received from the remaining authors.

